# Current and ceased users of sit stand workstations: a qualitative evaluation of ergonomics, safety and health factors within a workplace setting

**DOI:** 10.1186/s12889-018-6296-6

**Published:** 2018-12-14

**Authors:** Brendan Henderson, Rwth Stuckey, Tessa Keegel

**Affiliations:** 1 Centre for Ergonomics and Human Factors, School of Psychology and Public Health, College of Science, Health and Engineering, Latrobe University, Melbourne, Australia; 2 Monash Centre for Occupational and Environmental Health, Monash University, Melbourne, Australia

**Keywords:** Sit stand workstation, Ergonomics, Sustainability of interventions

## Abstract

**Background:**

Many workplaces have implemented sit-stand workstations (SSW), which enable a worker to transition between sitting and standing as they perform their work activities. The factors which determine the initial adoption, sustainability or cessation of use for a SSW, remain largely unexamined. This study investigates the experiences of workers who had previously used or were currently using a SSW.

**Methods:**

The study setting was within an Australian university. Participants who were current or past SSW users, as well as workplace key informants, were interviewed for the study. All interviews were recorded, transcribed and analysed. Transcripts were coded by two researchers for concepts and themes regarding uptake and sustainability of SSW. Discussion and validation of themes was undertaken by the team of three researchers.

**Results:**

A total of 24 interviews were conducted. Twenty-two interviews were with ceased and current users (16 current and six ceased users) and two interviews were with workplace key informants. Analysis of the interviews with current and ceased users identified three main themes: Personal considerations for use/sustainability; Posture; and Usability. Analysis of the interviews with key informants identified two themes: Considerations and concerns and Policies and procedures. Little information was provided to workers when first using a SSW. Workers who were able to adopt their working style to the new workstations were able to sustain ongoing use of a SSW. Key informants were concerned that employees believed using a SSW would provide a health benefit in its own right without an understanding of the possible risks that might be associated with use.

**Conclusions:**

Sustainable usage of this type of SSW is achievable, however, it requires some element of adaptation at the individual worker level. Participants spoke about how the use of the SSW in a standing position was typically associated with the time of day, specific task selection and musculoskeletal comfort or fatigue factors. The provision of education to new SSW users with relevant supporting information by a subject matter expert should enable the worker to obtain a more holistic understanding of the safety and health risks and benefits embedded in the use of a SSW.

**Electronic supplementary material:**

The online version of this article (10.1186/s12889-018-6296-6) contains supplementary material, which is available to authorized users.

## Background

Adult employees spend the majority of their day in a seated position, with research reporting that call centre workers in Europe can spend as much as 90% of their working day in a sedentary posture [1, 2]. It is also well recognised that the work environment is associated with an individual’s health and wellbeing status [3, 4]. Specific to the office work setting, there is a considerable body of evidence that suggests that there is an association between poor workstation setup and upper extremity body region pain and discomfort, which may increase an individual’s risk of musculoskeletal disorder when using a seated workstation environment [5, 6].

In recent years, many workplaces have implemented sit-stand workstations (SSW) which to varying degrees enable a worker to sit or stand as they choose whilst working [7, 8]. There are two common types of SSWs used, with the first one being a whole desk unit which is manually or power operated to adjust to the preferable height. The second option is a modular type desk top unit which sits on top of one’s desk and usually adjusted manually or with a set of lever locks. Literature suggests that a SSW can assist in reducing workplace sitting time, increase metabolic function and possibly reduce musculoskeletal disorder risk, for employees within the workplace [9–12]. It is also relatively unknown what impact a SSW has on work-related productivity [13, 14]. Whilst users have reported greater musculoskeletal comfort from height adjustable workstation use over a short term period [15], and there has been work to determine factors associated with sustainability of interventions after a workplace intervention [16, 17] factors associated with longer term in vivo use remain largely unknown. Much of the public health literature focusses on SSW users’ cardio-metabolic outcomes [18] as well as the role of organisational support and workplace culture in the uptake of SSW within the context of intervention trials [19]. However there is a lack of evidence around users’ understanding of their musculoskeletal disorder risk when seated compared to standing [20–22]. In addition to this within the literature there has been little examination of what factors determine the initial adoption, and ongoing use or cessation of use for a SSW within an actual (natural) workplace, as most published research to date has been restricted to introduction of SSW within short-term evaluation and research trial intervention environments [12, 23–25].

To address the gap in understanding of the experiences of both ongoing and ceased users of SSW in natural workplace environments, the purpose of this study is to investigate the systemic and ergonomic, safety and health related experiences of staff within a university workplace who had previously used, or are currently using, a SSW. A qualitative methodology was used to collect experiential data and provide insights into these key areas.

Finally, through encompassing an ergonomics approach, whereby the issue of adoption and ongoing use of a SSW is investigated using a wider systematic perspective, findings from this investigation will provide a greater understanding of the range of workplace factors which might influence SSW use, including organisational and environmental factors within real-life long term non-intervention trial settings [26] as compared to short-term controlled interventions.

## Methods

### Study setting and design

The study setting was a school within an Australian university which had a number of individuals using SSW. To be eligible for the study, participants must have previously used or be currently using a SSW within their workspace, be employed by, or studying within the university, and be aged between 18 and 65 years. More details regarding the study are available elsewhere [27]. Participants consisted of both staff and student researchers, and administrators, who performed varying amounts of their tasks at their desk. A current user was defined as a person who had adopted and undertaken continual use of a SSW for at least 3 months. To be defined as a ceased user, the participant was required to have used a SSW for a period of at least 3 months within their current role and had decided to cease using it. The types of SSW used by the participants in this study were either an Ergotron Workfit A, Ergotron Workfit S (Dongguan, China), or Standing Kangaroo model (Ohio, USA). These models can broadly be defined as pull-up, push down units, fitted as additions to existing workstations. The surface-space of these models is designed to only hold the monitor, keyboard and mouse, with limited room for other items or uses.

In order to better understand the barriers and enablers within the organisation regarding SSW use, two key informants were interviewed as part of the study [28]. The key informants were employed in positions which saw them undertake duties relative to the ergonomics, safety and health aspects of SSW implementation and use, for staff across the university.

This study was approved by the Latrobe University Human Research Ethics committee (S15/95), and all participants provided written informed consent for participation in the study.

### Users recruitment procedure

An email inviting their participation in the study, was sent to all staff and higher degree research students within a specific school of a Victorian university, which had a number of individuals using SSW. Key informants were identified in a phone discussion with the manager of the Health, Safety and Wellbeing unit within the university, and were directly approached and recruited into the study. Once recruited, all participants were asked to engage in a semi structured individual interview. The interview schedule is available as an online supplementary resource.

All interviews were undertaken face to face in the workplace by BH over a 6-week period between May and June of 2015, and were audio-recorded.

### Qualitative data collection

#### Individual current and ceased user interviews

Through a review of the existing literature [1, 2, 7, 8, 10, 20, 23, 25, 29–34], and an iterative discussion and development process within the research team, five domains of enquiry were developed within the interview schedule for the ceased or current users. These domains were: Reasons for using a SSW; Knowledge and understanding of ergonomic factors when using a SSW; Usability of SSW; Comfort when sitting and standing, and; Understanding of MSD risk. A semi-structured interview guide was developed and used to address each of these domains. See Additional file 1 provided online.

#### Key informant interviews

Utilizing a similar process of literature review [28, 32, 35] and development, nine domains of enquiry were proposed for the key informants’ interviews. These nine domains were: Policies and procedures regarding SSW; Issues surrounding SSW; Organisational barriers and enablers; Economic/ cost benefit analysis; Sourcing and installation of desks; Knowledge and understanding of ergonomic factors when using a SSW; Usability of SSW; Understanding of OHS risk, and; Understanding of MSD risk. Semi-structured interviews were used to address each of these domains.

### Qualitative data analysis

The qualitative research design made use of a thematic analysis approach [36, 37]. Participants were individually interviewed to capture the possible relationships, thoughts and opinions of both current and ceased SSW users [38]. During and following the interviews, field notes and observations were made by the interviewer to note relevant contextual and other observations to inform the analysis and study outcomes [39].

The interview audio recordings were transcribed by an external provider, with the lead researcher reviewing transcripts against the audio recordings to check for accuracy [36]. In order to establish the common factors and themes amongst the participants, specialised qualitative analysis coding software (NVIVO version 10), was used [40]. A sample of the interviews were reviewed and separately coded by another member of the research team. The research team had numerous meetings to discuss and refine the potential themes arising from the research data [36]. From this process, final agreement on the naming and relevant definition of categories and themes took place so that detailed thematic maps could be produced.

## Results

### Participants

A total of 24 participants (10 male and 14 female), comprising 22 current and ceased users, and two key informants (one male one female), volunteered to participate in the study. An outline of the SSW users by current or ceased use status is provided in Table 1. The key informants were employed in occupational health and safety roles which encompassed duties related to SSW requests and implementation.

**Table 1:** Current and ceased users demographic information

	Current (*n* = 16)	Ceased (*n* = 6)
Gender		
Male	4	5
Female	12	1
Age range (years)		
25–34	5	3
35–44	7	1
45–54	3	1
55–64	1	1
Occupational role		
Administration	3	1
Lecturer or above	10	4
Researcher	1	1
Research degree student	2	0
Length of SSW use Median (range)	21 months	15 months
	5–48 months	3–24 months
Employment arrangements		
Full time (1.0)	11	5
Part time (0.5 to 0.8)	5	1

### Qualitative data: SSW current and ceased users

Analysis of the current and ceased SSW user interviews identified a number of initial categories, themes and sub themes. Personal considerations for use/sustainability; Posture; and Usability were the final three themes identified from analysis of the interviews.

### Personal considerations for use/sustainability

All participants provided reflections on ‘personal considerations related to use/sustainability’ of a SSW. When asked about their reasons for wanting to commence using a SSW, current and ceased users provided a number of similar responses. Many reported that they had experienced pain or discomfort from sitting at work and wanted to improve their posture, stand more, and decrease their time spent sitting in the workplace. Many had the long-term goal of improving their health in some capacity and felt that a SSW could provide support for this. Participants provided their knowledge and understanding about the perceived health benefits of using a SSW. Many saw the use of SSW as socially desirable and sustainable due to peer support as well as support from management to use a SSW.

*Basically I know that sitting all day is not good for you and so when the opportunity presented a couple of years back to get a sit-stand station I thought, well, it can't hurt to have one. At least then I've got the option to not sit. That was pretty much the reason. It wasn't that I found that I was in pain or discomfort or anything sitting for long periods. I just knew that it wasn't good for me so when you've got the option to do something about it; do it.* Participant 11 (current user).

*I’ve heard that sitting is bad for you, and I feel like a hypocrite telling people to stand up if I don’t. Obviously just working in this kind of an environment you kind of - all the bigwigs in the corridor promote standing so we’re just fitting in with culture I guess.* Participant 9 (current user).

Those who had ceased using a SSW had similar reasons regarding their decision to start using one.

*Just to try it and see if it would be a useful way for me to not sit down as much given all the propaganda, hype, information… energy expenditure and health.* Participant 16 (ceased user).

Ceased users offered a variety of reasons ranging from why their usage gradually lessened over the day, to perceived lack of efficiency/productivity, for not persisting with the SSW.

*….there’s a component of it just sort of fading away and me sitting more, and not really thinking about it.* Participant 19 (ceased user).

*I felt that I was far less efficient standing. Again it sounds really odd but I just felt like I couldn’t concentrate well enough. Maybe I just feel better when I’ve got a lot more space, and so having the model that I had, it didn’t have a lot of desk space as such, so I couldn’t spread out my gear.* Participant 3 (ceased user).

Participants discussed the design and usability of the SSW, the time of day and the complexity of the tasks being undertaken, their ability to make decisions regarding when to undertake work in a standing position, and their comfort and fatigue levels.

Most current users considered the time of day when considering undertaking work in a standing position. Participants generally spoke about a preference for standing in the morning. Many users discussed a decline in standing as the day progressed.

*I tend to find that I use it first thing in the morning as soon as I get here. But I tend to - I probably use it half and half. So over the course of an entire day there's just periods where I stand and periods where I sit. So I wouldn't stand all morning or stand all afternoon or anything like that.* Participant 11 (current user).

Task selection by both current and ceased users was a key consideration in whether one would use the desk in a standing position. Many communicated that their preference was to work whilst standing for tasks which required a lower level of concentration or where workflow was not impeded by the space constrictions of the standing workstation.

*I prefer to use it by task. I don't find it very good for writing and editing work… I don't have enough space around me for my paper and other things, so I find it really good for obviously, video conferences, emails, tasks where I don't need to refer to other publications or something. But I find for editing tasks - real thinking tasks where I need to draw on other resources, I sit.* Participant 15 (current user).

*The only time I really used it for standing is when I didn’t need to think in a lot of detail, which seems really silly. But I found that I couldn’t concentrate very well when I was standing to use it. But also if I needed any other materials. So if I needed to read off a document or hard copies of things, then it wasn’t useful because there was limited space.* Participant 3 (ceased user).

In terms of productivity, participants’ views were mixed with no clear indication of decreasing or increasing productivity when using the desk in a standing position.

*I think productivity would be lower, because it’s inconvenient you know to have to change heights to be able to do different tasks, or be able to read different pieces of paper. A lot of the tasks were neutral yeah, if it was just standing stuff, that I didn’t need to refer to pages that were lower down, then it didn’t matter.* Participant 18 (ceased user).

Many of the users reported an awareness of their own comfort and fatigue in both standing and sitting postures despite most users reporting that they have never received or sought to understand basic ergonomic and workplace injury risk principles. Postural awareness was mentioned by a few participants which suggests that some have an understanding of appropriate office ergonomics and safe workstation use.

*….after a period of time my feet get sore. Then I get back to a sitting position and I sit for longer than I should and then I remind myself again that I need to get back up into a standing position to do something.* Participant 6 (current user).

A number of the female participants reported footwear selection being a key consideration for enabling work to be undertaken standing. This illustrates that participants, not only value working in comfort and possibly understand the implications on their body if they choose to wear what they consider inappropriate footwear, but that they are also willing to implement a behavioural change to ensure they can work in a standing position.

*Sometimes it can be a little bit uncomfortable just on the soles of my feet. I typically now wear flat shows because it's pretty uncomfortable to stand in high heels all day, or any kind of heel actually. The other thing I do is take my shoes off sometimes, because it's more comfortable to stand in bare feet. So I think it makes me choose my shoes differently, because I think standing in any kind of heel all day is not comfortable at all.* Participant 20 (current user).

Elements of ergonomics, safety and health relative to SSW were mentioned both directly and/or indirectly. Many of the users spoke about positive associations with their health and wellbeing status through using a SSW.

*Because of the ergonomics, because human beings aren't meant to sit as far as I'm concerned. The spine's not designed for sitting; it's designed for standing and walking. People tend to get into bad postures when they're sitting. It's just the nature of the beast and I'm sitting now and my shoulders slumped forward and arched back. The body's not designed to sit.* Participant 21 (current user).

Overall, users were quite forthcoming about their personal considerations for sustained use or not. Other factors discussed by some participants included the positive psychosocial associations in using the workstation, cognitive demands and mood status.

### Posture

Associated with the personal considerations for use, participants discussed a variety of factors associated with their comfort and kinaesthetic awareness. These have all been brought together under the theme of ‘posture’. To ensure ongoing feasibility of use, many mentioned behavioural change aspects such as being dressed comfortably, but also the physical element of having good body positioning so they could undertake their tasks in comfortable standing posture at their discretion. Participants discussed the reasons they would typically change back to working in a seated position as primarily being related to comfort and fatigue.

*I think it’s just - it’s just postural I guess. I do tend to hunch a lot when I sit. So it’s just me being conscious about my body getting into that position that prompts me to want to stand up a little bit more. The upper back, upper mid-back I suppose, your shoulders as well as around the neck.* Participant 17 (current user).

Many of the participants made mention of moving whilst standing with reference to discomfort from static standing and the potential of this becoming a health issue.

*I would say that I plant the feet and stand in that one position, then basically after a little while, when it gets sore, I'll move my feet around and come back to that position.* Participant 6 (current user).

The opinions of users regarding their musculoskeletal disorder risk when sitting or standing was somewhat varied, however there was a general sense that their risk of injury was higher when working in a seated posture. Participants commented that the risks were highest for the neck, shoulder and back regions when sitting.

*Probably back, I guess, just from a postural perspective, you know, sitting all day can't be - it can't be good for you. And, yeah, I'd say probably back, maybe neck depending on what you're doing, you know, if you're leaning over your desk or writing all day kind of scenario.* Participant 11 (current user).

When adopting a standing posture, participants spoke about their knowledge and understanding that there might also be musculoskeletal disorder risks present to areas of the lower body. A lack of understanding of what some of the risks are was evident within a number of the responses.

*I’m not really sure what the risks are. Certainly in my experience it's been probably pooling of blood in your calves and a bit of aching around your feet and ankles. But that’s all I really notice and I guess there’s probably some risks to standing too much as well, perhaps lower back pain for some people, but it certainly hasn’t been something that I’ve experienced.* Participant 20 (current user).

Those who had ceased use of their SSW, provided various reasons as to why this was the case. Some commented on anthropometric issues such as the uppermost height of the workstation not being adequate for accommodating their personal use. Others questioned if there was value in using the SSW if static standing was adopted when operating in the standing position.

*I also have issues with how good standing in one place for a long period of time is for people in terms of health. I’m not too sure that that’s an ideal alternative to sitting, which is basically what you have to do at a standing desk. I mean you can shuffle around a bit but - and I think in the past, probably in days gone by, people who had jobs where they had to stand a lot or move around, nurses and waitresses and things like that, often used to have issues with the lower limbs. That seems to - I don’t know where that’s gone these days, but I still think that maybe static standing itself may not be ideal.* Participant 19 (ceased user).

Other factors related to comfort included the effect that a warmer day might have on their time spent standing, and the use of ergonomic office aids such as a keyboard support providing assistance with comfort levels within their workspace.

### Usability

Most participants provided lengthy commentary regarding the ‘usability’ of the workstations. Nearly all interviewees confirmed that they had never received any personalised formal training upon the installation and setup of the sit stand workstation.

*None. Not in regards to getting given a standing desk.* Participant 1 (ceased user).

*Yeah, we’ve done basic stuff in part of the induction when we first started the job but that was it. There’s this stuff that’s floating around on the noticeboards…* Participant 22 (ceased user).

Some participants reported having a basic level of knowledge and understanding of the key ergonomic considerations of using a SSW. There were a low number of participants who had not sought to understand the requirements or key ergonomic principles linked to a safe workspace in order to mitigate their exposure to musculoskeletal hazards through either internal or external resources. A small number of participants had spoken about undertaking internet searches to upskill their knowledge and understanding.

*I’ve never really thought about it. I guess I’m satisfied with my knowledge but in saying that I’m sure there’s probably a lot more that I might need to know.* Participant 12 (current user).

*I thought I did but probably - well it’s probably really never been high on my list of concerns…. I’ve received no formal training in how to use a standing desk and maybe I should have….we were just asked did we want one, and they were installed, and that’s pretty much it. Not that they’re very technical or challenging to use, but you just kind of make the assumption that you know how to use it.* Participant 2 (current user).

When it came to setting up their workspaces and using the SSW, a number of users ensured they took their comfort into account.

*Haven’t been given any training at all or any advice about using the desk, I’ve just used my own common sense. If I feel uncomfortable I sit down. I know that standing up all the time is not a great thing either. So I just use my own common sense and listen to my body. But I haven’t actually been provided with any formal training or anything whatsoever.* Participant 20 (current user).

*I just go with comfort like whatever feels comfortable I think. But there's no - I can't think of any particular way I would set it up.* Participant 4 (current user).

Aspects of adaptation to a workspace were spoken about by current users, typically to improve functionality. Some current users were willing to modify their work behaviours and forgo some functionality of their workspace so they could continue to use their SSW. Many users believed that the positives of having the option to be able to operate across a working day between a seated or standing position, outweighed the negative of the loss of a fully functional workspace.

*I find standing is fine, but it’s this style of desk whereby, as I said, you have nowhere to put paper at eye-level or even reasonably close. It's got to be down on the desk so I don't find that useful but whereas I've seen different standing desks that actually have space for you to put your paperwork, which is near your keyboard, and so it looks like it's a more useable kind of set-up. But I don't go home from work every day feeling uncomfortable. If I did that I wouldn't use it in the way that I use it. So it works for me but it's - I know that there are better systems than what I've got and the ultimate would be to have an actual desk that moves up and down.* Participant 4 (current user).

Of the ceased users, it was reported by some that the lack of usability and loss of workspace were key factors in their decision to stop using the SSW and removing it from their workspace.

*No that’s why I got rid of it. It was just taking up so much space and even when I had it in the lower position to sit, you still had the keyboard in the way and it was just a nuisance. The particular model I had I thought it was easy enough to lift to adjust the height of the unit, but just the space it gave you in regards to having – even where you placed your mouse in regards to the keyboard or any documents that you needed were a bit of a challenge.* Participant 3 (ceased user).

Nearly all users conveyed both positive and negative factors regarding their evaluation of the workspace modifications required to ensure they could undertake their work.

*Things fall off the side. There's not enough room for my documents right next to where I'm typing, and I don't like it because it's not positioned well on my desk for where I need to stand.* Participant 15 (current user).

Other factors which participants raised included furniture placement within the workspace, the functionality of the modular style workstations, and the lack of a subject matter expert to ensure that their workspace was safe and ergonomically friendly for use. Current users also expressed concerns about having an optimum setup for their workspace, so use could be ongoing and sustainable, to address issues including accessories such as adequate cable lengths, additional furniture placement and overall functionality of the workspace.

### Key informant findings

Upon analysis of the two key informant interviews, these were found to encompass two main themes: Considerations and concerns, and Policies and procedures.

### Considerations and concerns

The key informants spoke about the ‘considerations and concerns’ around the lack of education and understanding of employees. Key informants were concerned that employees believed using a SSW would provide a health benefit in its own right without an understanding of the possible risks that might be associated with use.

*First of all, I would ask the question why, why they need one and why they would like one. If it is because they've had stated issues with their work situation, I'd ask to go and see if their workstation setup is adequate because sometimes there'll be something that's out of whack that might be causing an issue for them.* (Key informant 2 -Manager of Health Safety and Environment (Faculty level).

Concerns were raised regarding risks within a work environment when a sit stand workstation was implemented including ensuring the workspace was not impeded and that users were not adopting a static standing posture.

*There’s problems with static standing…it can be very fatiguing, so it’s almost like well I’ve got it, now I’m going to stand here for 8 h a day. Well that’s not what they’re designed for, and I think that’s potentially one of the things where people - it could create a problem where there wasn’t one before if people aren’t using it properly, so that’s probably one of the risks. In terms of other OHS risks hopefully the risk assessment we do would eliminate those risks, and so by the time people get to use it they understand that we’re suggesting that they sit and then stand, and then sit and change their posture during the course of the day.* (Key informant 1 – Senior OHS consultant).

### Policies and procedures

Discussions around the ‘policies and procedures’ in place highlighted that whilst the key informants provided advice and training to employees in regards to SSW, it was not their final call in some instances to give approval for an employee to install and use the workstation, as financial approval for the institution is located within each work group.

*I certainly don’t receive all requests for sit stand workstations. If there is no underlying medical condition, health condition or disability, it really is up to the local area as to whether they purchase it. They hold the budget for it. If a request comes to me though we do an assessment, we obtain some medical information so that we understand what the person’s condition is. And then we’ll make recommendations back to the treating practitioner about what we think is suitable. You know we would sort of have a dialogue between - with the staff member and their practitioner, and we’d pick something that was suitable.* (Key informant 1 – Senior OHS consultant).

Finally, it was mentioned that staff have access to additional workplace health and wellbeing options that can assist to establish a behaviour change to improve one’s health and wellbeing status.

*I think it’s great to have the management on board, and sort of driving the issue and understanding that it could have benefits. Listening to what people are saying, but also talking them through the practicalities because it’s one strategy in a whole suite of strategies around keeping people fit and healthy at work. It’s one thing. And I don’t think we can over-focus on it, we’re currently working on our Health and Wellbeing Program and trying to establish a very significant physical health program for our organisation. And the sit stand workstation addresses one thing around sedentary work, but we have other strategies that we can work on as well.* (Key informant 1 – Senior OHS consultant).

## Discussion

This study provides a qualitative assessment of the ergonomics, safety and health factors associated both with the longer-term sustainability of use, as well as the cessation of use, for SSW within a workplace environment. Most previous qualitative studies of SSW use have been conducted as short term evaluation components of larger research studies, which either sought to implement SSW in workplaces in a systematic way, or were conducted within a laboratory/ simulated workplace environment [11, 12, 25, 30]. Evidence from these studies has shown positive health effects as well as the importance of organisational support and workplace and individual culture in the successful adoption of SSW [16, 19, 41]. The results of this study, in comparison, highlight the use of SSW within a natural workplace environment. In addition to this, important insights were also obtained from the small number of now ceased users who participated in the study. Our study also included interviews with key workplace informants, who were able to provide insights from a systematic perspective regarding organisational and environmental factors for the use of SSW. Another important contribution is that some participants within this study have been using the SSW for much longer than previously researched populations.

Our research also found that a level of social desirability and support from management was present in the workplace, with availability of health and wellbeing based initiatives, including the option of using a SSW, if staff wished to utilise them. This is similar to findings from a previous qualitative evaluation of SSW in an Australian workplace [6, 25]. Surprisingly, most participants in our study did not specifically discuss sedentary behaviour and the development of cardio metabolic risk factors [42], which have been strongly publicised within the media. This could possibly be due to a lack of user understanding about these associations, as this group of workers were not provided with any formalised education regarding the potential health benefits of using a SSW upon installation or at the time they were offered the SSW option.

Throughout the interviews, discomfort mitigation, along with positive health and postural improvements, were raised by participants as reasons for initial uptake and use of a SSW. Participants’ reasons for adoption and use were generally related to the perceived musculoskeletal health benefits of operating a SSW. This finding is similar to previous research which saw workers report similar health reasons for wanting to use a SSW [30]. Participants who ceased use of SSW identified the lack of functionality within these types of units as either the workspace not being large enough for the work being undertaken, or taking up too much space on the supporting desk. Our findings are also novel in establishing a number of considerations that current SSW users put in place to ensure ongoing sustainable use within their workplace.

A range of ideas emerged from the data, which were mapped into three main themes for the current and ceased users: 1) Personal considerations for use/sustainability; 2) posture; and 3) usability; and two themes for the key informants: 1) considerations and concerns; and 2) policies and procedures. Themes for current and ceased users, as well as key informants, are discussed in detail below.

### Current and ceased SSW users: Personal considerations for use/sustainability

The findings highlight a number of notable personal considerations for use/sustainability of a SSW, in addition to other personal adaptations implemented over a longer term usage period. It was evident that many users had a preference for using the workstation in a standing position in the early part of their workday and for tasks that were considered lower in complexity. Chau et al. [30], reported similar findings over a 4-week intervention period, with participant’s usage patterns grouped into being either task based, time based or having no particular pattern. Of the females interviewed in our study, nearly all discussed the need to be wearing comfortable footwear in the workplace so that they could work whilst standing. Although previous research also touches upon this preference [23], our findings revealed that many of the female participants actually took into consideration the need to plan wearing comfortable footwear in order to adopt a standing position in the workplace.

Although productivity was not quantitatively assessed, participants were asked to provide their opinion about any perceived changes to their productivity since the adoption of using a modular SSW. Participant responses were mixed. Task selection was widely discussed with many users being selective about what tasks they would undertake in a standing position. This supports previous research questioning if the use of a SSW resulted in increased worker productivity due to the level of variation and the ability for participants to select their use of the workstation in a seated or standing position [10, 12].

Our findings related to longer term sustainable use provided some similarities to those reported in a pilot study by Alkhajah et al. [23], who found that self-reported health and work performance outcomes did not change markedly after 3 months of SSW use. Whilst our findings saw longer term use, with a mean of 21.7 months, users indicate that some level of adaptation has occurred to their workspace so that sustainable use is possible, a number of users provided insights into being rather selective relative to what tasks are undertaken in a standing positon. This demonstrates that users have been able to implement a protocol of using a modular SSW to suit their individual needs so that sustainable use can occur. However, this does come with some adaptation required, particularly in the areas of a loss in available surface space and work task selection, at the expense of the individual.

Previous research has found that when workers receive training or instruction from a subject matter expert such as an ergonomist, they were nearly twice as likely to use the SSW on a daily basis [34]. In our study it was evident from the responses that participants received very minimal guidance or training on what is considered a safe and ergonomically friendly workspace when using a SSW. Most participants, however, generally conveyed that they felt that they were capable of setting up the workspace appropriately so they could undertake their tasks in either a seated or standing position. Even though the study participants mostly felt competent to arrange their SSW, our study findings suggest that more can and should be done to prepare employees for initial safe operating use of a SSW [43]. As such, the need for selection of a more functionally appropriate workspace design, whereby staff are adequately educated on all aspects of their workspaces through a subject matter expert, is important. This may assist future successful implementation and utilisation of SSW [10].

### Current and ceased SSW users: Posture

While participants reported being aware of their posture and comfort, a lesser number appeared to understand the possible risks of prolonged standing or static posture. Upon weighing up whether a seated or standing position carried a higher level of musculoskeletal disorder risk, the majority of users stated that being in a seated position posed a higher risk, to the lower back, neck and shoulder regions in particular, compared to the risk to the lower limbs when operating in a standing posture. Users adopted various methods within their workspace to ensure that their posture was functional and comfortable for the task being performed. Aspects of behaviour change were discussed relative to the domains of comfort, body awareness and positioning, standing and sitting posture ratios [44]. All of these are deemed to be critical elements of a safe and ergonomically friendly workspace [8, 29, 45].

As discussed earlier, longer term users spoke about the duration they operated in a standing position across a working day. Whilst this varied between those interviewed, no user formally adopted a ratio of sitting to standing with their workstation, although many performed approximately half their working day in the standing position. Many discussed the requirement to build up a level of conditioning to the lower limbs by initially increasing the standing time at the early stages of use. Literature within the static sitting domain confirms that people who break up their sitting time with time spent standing can improve their fatigue resistance across a working day [46, 47]. It is plausible to suggest that the implementation of a gradual increase of time spent standing will aid lower limb conditioning, and that this should be encouraged [17]. Further to this, a set of universal guidelines which encompasses a ratio of sitting to standing based work could be implemented to provide guidance for users [11].

### Current and ceased SSW users: Usability

With the immediate interface between the user and the equipment being very important, the usability and workspace layout are critical factors to ensure a level of worker acceptability and functionality [31]. A number of SSW users in the study were willing to adapt their working conditions without consideration of the possible negative biomechanical and ergonomic implications to a functional workspace. This may have given rise to risks for musculoskeletal discomfort and injury [17], which has possible implications in regards to the task design and workflow factors that many study-participants, including most ceased users, discussed as a limitation of the workspace. These findings are supported by a previous study which reported many of their cohort who trialled pull-up push down workstations, like the ones which were used in this study, had issues around the ergonomics factors of the workstation design and their impediment to comfort, stability, anthropometry and loss of functional workspace [30]. These factors can ultimately affect an individual’s safety and health [48, 49] and are important to consider with the workplace implementation of all SSW designs, both pull-up, push down designs as well as single surface SSW in which the entire work desk surface is raised and lowered.

Previous research has highlighted that individuals will consider the possible benefits and issues associated with losing part of, or needing to modify their workspace so continuation of use can occur [25, 34]. In regards to the ceased users, the loss of functional workspace due to the design and modified workspace layout was a key consideration with usage cessation. Similar issues were raised by participants who used SSW over a short term period, highlighting the need for a holistic assessment before implementation [23, 30].

### Key informants

The key informants discussed related issues regarding the ergonomics, safety and health associations with a SSW. They were well informed in regards to their knowledge base of SSW use, which supports recent findings of a study of occupational health and safety practitioners who discussed the health risks, intervention strategies and influences related to occupational sitting [32]. The key informants’ views were that a systematic approach is required with the implementation of SSW, and that this approach should address considerations and concerns, as well as relevant policies and procedures so that an informed decision can be made by prospective SSW users [35].

### Study strengths and limitations

As described above, all of the users and ceased users within this study were provided with pull-up push-down style workstations, and as such some of the study generalizability may be limited to other organizations which have implemented this style of SSW. However other study findings, particularly with respect to education at the time of initial SSW implementation, are more broadly generalizable. Another limitation is that this study did not collect detailed information about socio-economic factors. The interviewees were a mix of administrative and academics at a university and as such there is less variability in socio-economic factors compared to the general population. Our study has some notable strengths in that the majority of the workers who participated had used the workstations for more than 3 months and were ongoing SSW users within their natural work setting. None of our study participants were introduced to the SSW in their current workplace as part of a research trial and as such this study provides important information regarding the implementation, use and sustainability of SSW in real (natural) workplace settings. Valuable insights were also obtained from the small number (*n* = 6) of participants who had ceased use. Future research with larger numbers would be useful to enhance this preliminary assessment of ceased SSW users. Another area for future research is looking at the long-term comparison of different types of SSW. Finally, inclusion of the views of the key informants are also an important strength of this study, because it is individuals in the workplace such as the key informants, who may be responsible for future workplace SSW implementation as well as the development of SSW workplace policies.

## Conclusions

In conclusion, this study has provided valuable insights into the ergonomics, safety and health domains relative to longer term SSW use within a natural work environment. A number of important personal, postural and usability factors have also been identified relative to ongoing and sustainable SSW use. Participants stated that the adoption and use of a SSW would provide health benefits, and they spoke about how the use of the SSW in a standing position was typically associated with the time of day, specific task selection and musculoskeletal comfort or fatigue factors. The study findings have practical relevance to organisations who are looking to implement SSW for employees, particularly in regards to worker education at the time of introducing SSW to a workplace. This study found that sustainable usage of this type of SSW is achievable, however, it most likely comes with some element of adaptation at the individual worker level. The provision of education to new SSW users with relevant supporting information by a subject matter expert such as an ergonomist, should enable the worker to obtain a more holistic understanding of the ergonomics, safety and health risks and benefits embedded in the use of a SSW.

## Additional file

Additional file 1: Interview schedule. Interview schedule used for the qualitative interviews with ceased and current sit stand desk users. (DOCX 99 kb)

## Abbreviations

MSD: Musculoskeletal disorder
OHS: Occupational health and safety
SSW: Sit stand workstation
