# Supplementary material for: Current and ceased users of sit stand workstations: a qualitative evaluation of ergonomics, safety and health factors within a workplace setting
Source: BMC Public Health. 2018 Dec 14;18:1374. doi: 10.1186/s12889-018-6296-6 (PMC6295098; doi:10.1186/s12889-018-6296-6)
Supplement: Supplementary file 1 — Interview schedule. Interview schedule used for the qualitative interviews with ceased and current sit stand desk users. (DOCX 99 kb) [file 12889_2018_6296_MOESM1_ESM.docx]

# Interview schedule for ceased and current sit stand desk users

| Area | Question | Notes |
| --- | --- | --- |
| **0 – Background information** | What age or age bracket are you within?  What gender are you? What is your role?  What is your time fraction?  How long have you been using a SSW for? | **Explain method- audio or document record**  **Oral interview or questionnaire** |
|  | Can you tell me if you have experienced any discomfort, pain or injury in following regions before using the SSW - neck, shoulder, upper back, lower back, lower limbs, upper arm, elbow, forearm, wrist and hand |  |
|  | Can you tell me if you have experienced any discomfort, pain or injury in following regions since the implementation of using the SSW - neck, shoulder, upper back, lower back, lower limbs, upper arm, elbow, forearm, wrist and hand |  |
|  | What type of SSW do you use? |  |
|  | What is a typical week for you at work in terms of time spent undertaking:  Sitting? Standing? Walking?  Heavy labour or physical demanding tasks? |  |
|  | Workforce Sitting Questionnaire |  |
|  | Can you point to me on this diagram where you have felt pain and discomfort prior to using a SSW? | 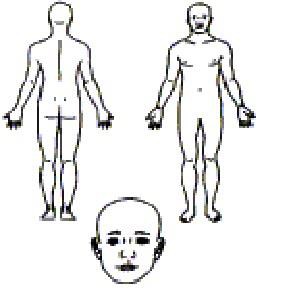 |
| **1 – Reason for using a SSW** | Tell me your reasons for using a SSW (SSW)? |  |
|  | What do you think are the benefits of using a SSW? |  |
|  | Would you say you use it on a |  |
|  | regular basis? Yes/No Daily?  How many times a week? Is it task dependant? |  |
|  | How do you typically use your desk? Ie –do you use it for 20 minutes 5 times a day, or one hour once a day etc…. |  |
|  | *If a ceased user, why did you stop using a SSW? e.g. - discomfort, pain, lack of functionality?* |  |
| **2 – Knowledge and understanding of ergonomic factors when using a SSW** | Can you provide any insight to me about any training provided to you on how to use your SSW, and how to transition between the two workspaces once the SSW was installed?  Yes- what did the training involve? No – next question |  |
|  | Have you ever been provided with training on basic ergonomic principles of a good workstation setup when:  A – Sitting?  B – Standing? |  |
|  | If so, what were you the key points communicated to you for each of these positions when undertaking your work:  A – Sitting?  B – Standing? |  |
|  | If you have never undertaken basic ergonomic training for your workspace, have you seeked to understand the requirements through other means such as the internet, organisation briefs, etc.? If yes, what were these methods? |  |
|  | Do you feel that the setup of a sitting workstation and a standing workstation are similar?  If yes, why so? If no, why not? |  |
|  | Do you feel comfortable with your level of knowledge regarding the safe use, transition and setup of a SSW and why? |  |
| **3 – Usability** | Describe to me the usability of your SSW? |  |
|  | What do like about using a SSW from a workspace perspective? |  |
|  | And your dislikes? |  |
| Do you find it easy to transfer from a sitting to a standing workstation setup or vice versa? | | |
| What factors do you take into consideration when moving from a sitting to a standing desk setup and vice versa | | |
| **4 – Comfort** Do you ever find comfort is an issue when operating in a standing position? | | |
| Do you experience discomfort or pain when sitting for a prolonged period?  If yes, what regions of the body? | | |
| Do you experience discomfort or pain when standing for prolonged period?  If yes, what regions of the body? | | |
| Do you use any ergonomic aids when using your SSW- such as gel mouse pad, anti-fatigue matting?  If yes, why? | | |
| **5 – Understanding of** Tell me what you believe to be the **MSD risk** main risks to your body when working:   - Seated? - In a standing position? | | |
| Do you feel your level of MSD risk Explain what MSD risk is. when working is higher when  seated compared to standing, or vice-versa?  Can you provide me with your thoughts and reasons as to why or why not? | | |
| **Closing remarks** In closing, is there anything else  you would like to say about your experience using a SSW or | | |

# Interview schedule for key informants

| Area | Question | Notes |
| --- | --- | --- |
| 0 – Background information | What is your role?  How much of your typical working week is taken up addressing the SSW area? |  |
| 1 - Policies and procedures regarding SSW’s | Does the university have a formal policy regarding SSW’s?  If yes, what does it entail?  If no, is there anything such as guidance notes or procedures available for staff and areas to view regarding the SSW topic? |  |
|  | On what grounds is a worker within the organisation provided with a SSW? |  |
|  | Who financially supports the purchase and installation of a SSW? |  |
| 2 – Issues surrounding SSW’s – including MSD risk | What do you see the issues surrounding the use of SSW’s? |  |
|  | Any other issues to add? |  |
|  | What do you believe are the positive benefits associated with workers using a SSW? |  |
|  | And the negatives associated with workers using a SSW? |  |
| 3 – Organisational barriers and enablers | What do you feel are the barriers of implementing SSW’s within the organisation? |  |
|  | What do you feel are the enablers of implementing SSW’s within the organisation? |  |
| 4 – Economic/cost benefit analysis | Do you believe there is an economic cost benefit to be had from the installation and use of a SSW?  If so, where do you think the costs benefits are?  If not, why? |  |
| 5 – Sourcing and installation of SSW’s | Is there a certain type of SSW you recommend? If so, why? |  |
| 6 – knowledge and understanding of  ergonomic factors when using a SSW | Does the workers workspace come into consideration when  advising on the implementation of a SSW? |  |
|  | Do you have to provide additional training to users of SSWs? |  |
|  | What do you believe are the reasons for staff wanting to use a SSW? |  |
| 7 - Usability of a SSW | Have you found that SSW users are reporting back to you their perceived benefits or negativity associated with using a SSW?  If so, what is the feedback? |  |
|  | Returning back to sitting workstation setup? |  |
| 8 - Understanding of OHS risk | What do you see are the OHS risks with a person using a SSW? |  |
|  | Do you believe that a SSW poses a greater OHS risk compared to a standalone, sitting or standing workstation?  Can you explain your reasons behind this? |  |
|  | How are these risks within the organisation mitigated? |  |
| 9 - Understanding of MSD risk | Do you believe that the use of a SSW can mitigate MSD risk within a worker?  If so, what particular MSD risks? If not, why? |  |
|  | Do you feel that users are somewhat sold of the benefits of a SSW without an understanding of the current research literature available? |  |
| Closing remarks | In closing, is there anything else you would like to say about the SSW area? |  |
